# PSMA-homing dsRNA chimeric protein vector kills prostate cancer cells and activates anti-tumor bystander responses

**DOI:** 10.18632/oncotarget.15733

**Published:** 2017-02-25

**Authors:** Yael Langut, Nufar Edinger, Efrat Flashner-Abramson, Naomi Melamed-Book, Mario Lebendiker, Yael Levi-Kalisman, Shoshana Klein, Alexander Levitzki

**Affiliations:** ^1^ Department of Biological Chemistry, Unit of Cellular Signaling, Silberman Institute of Life Sciences, Safra Campus, The Hebrew University of Jerusalem, Jerusalem, Israel; ^2^ Department of Biological Chemistry, Unit of Bio-Imaging, Silberman Institute of Life Sciences, Safra Campus, The Hebrew University of Jerusalem, Jerusalem, Israel; ^3^ The Protein Purification Facility, Wolfson Center for Applied Structural Biology, Silberman Institute of Life Sciences, Safra Campus, The Hebrew University of Jerusalem, Jerusalem, Israel; ^4^ The Center for Nanoscience and Nanotechnology, Silberman Institute for Life Sciences, Safra Campus, The Hebrew University of Jerusalem, Jerusalem, Israel

**Keywords:** PSMA, polyIC, dsRNA binding domain, ScFvJ591

## Abstract

The treatment of metastatic androgen-resistant prostate cancer remains a challenge. We describe a protein vector that selectively delivers synthetic dsRNA, polyinosinic/polycytidylic acid (polyIC), to prostate tumors by targeting prostate specific membrane antigen (PSMA), which is overexpressed on the surface of prostate cancer cells.

The chimeric protein is built from the double stranded RNA (dsRNA) binding domain of PKR tethered to a single chain anti-PSMA antibody. When complexed with polyIC, the chimera demonstrates selective and efficient killing of prostate cancer cells. The treatment causes the targeted cancer cells to undergo apoptosis and to secrete toxic cytokines. In a bystander effect, these cytokines kill neighboring cancer cells that do not necessarily overexpress PSMA, and activate immune cells that enhance the killing effect. The strong effects of the targeted polyIC are demonstrated on both 2D cell cultures and 3D tumor spheroids.

## INTRODUCTION

Prostate cancer is the second most commonly diagnosed cancer worldwide, accounting for over 25% of new cancer cases diagnosed annually among men in the US [1]. Patients with metastatic prostate cancer are usually treated with androgen deprivation therapy (ADT). While ADT generally achieves short-term remission, patients typically develop castration-resistant prostate cancer (CRPC). There is a great demand for novel therapies for CRPC patients, as these patients rarely respond to existing therapies and demonstrate median survival of about 3 years [2–4].

Current targeted cancer therapies delay, but rarely prevent, tumor progression. As tumor cells are genomically unstable, they eventually acquire mutations and genetic alterations that allow them to evade the therapy and develop resistance [5–7]. The rate of killing elicited by targeted agents is too slow, allowing the tumors to adapt to the therapy [8–10]. Additionally, tumors are heterogeneous and possess a number of different subpopulations [11–13]. Targeted therapies are directed at specific subpopulations, and therefore cannot be expected to eradicate the entire tumor. We believe that an effective targeted therapy must kill the multiple sub-populations of the tumor simultaneously and rapidly, to preclude the development of resistance.

We have recently developed a new and promising strategy to develop such anti-cancer agents. Our strategy is to deliver synthetic dsRNA, polyinosine/polycytosine acid (polyIC), specifically into cancer cells. In cells, polyIC mimics dsRNA viruses, invoking anti-viral defense systems that cause cellular apoptosis and trigger an innate immune response. Thus, specific delivery of polyIC into cancer cells leads to massive killing of the targeted cancer cells, and provokes an innate immune response against neighboring, untargeted cancer cells. The rapid destruction of tumors and the concurrent use of multiple killing pathways increase treatment robustness and impede the development of drug resistance. Specific delivery of polyIC is achieved by attaching polyIC molecules to a targeting ligand, which homes to a receptor that is overexpressed on the surface of the cancer cell. Receptor overexpression provides treatment selectivity, by discriminating between cancer and non-cancer cells.

The secretion of cytokines and chemokines in response to the presence of polyIC leads to “bystander effects”, i.e. the killing of neighboring untargeted tumor cells. Thus, the treatment is potent even against heterogeneous tumors [14]. We define two types of bystander effects: (1) A *direct bystander effect*, whereby toxic cytokines secreted from the targeted cancer cells lead to the activation of apoptotic pathways in neighboring, untargeted cancer cells; (2) An *immune-cell-mediated bystander effect*, whereby chemokines secreted from targeted cancer cells recruit and activate immune cells, enhancing the killing of neighboring, untargeted cancer cells.

Metastatic CRPC typically presents a unique cell surface molecule that can be exploited for targeted therapy: prostate-specific membrane antigen (PSMA). PSMA is overexpressed at levels of up to 1000-fold at all Gleason scores [15, 16], while overexpression increases with tumor progression [17, 18]. Despite the heterogeneous nature of the disease, primary tumors or metastases that are completely PSMA-negative are rare [19]. While the above findings support the notion that PSMA is a highly promising therapeutic target, no PSMA-targeted therapies are currently approved for clinical use. However, a few agents are in clinical trials [20–23]. PSMA behaves like a classical receptor, exhibiting rapid internalization and recycling upon ligand binding [24]. Thus, a therapeutic entity can be coupled to a PSMA ligand and internalized into prostate cancer cells. Indeed, a variety of PSMA-targeting ligands are being investigated for tumor imaging [25–27] and for the development of PSMA-targeted therapeutic agents [28–31].

In our laboratory we have developed chemical vectors to deliver polyIC to EGFR-overexpressing glioblastoma [32], breast cancer and vulval carcinoma [14], as well as HER-2-overexpressing breast cancer [33]. The targeted polyIC proved to be extremely efficient at killing tumors *in vitro* and *in vivo*, leading in some cases to complete tumor eradication [14, 32].

Herein we present a protein alternative, rather than a chemical vector, to target PolyIC to prostate cancer. A protein vector offers multiple advantages over the use of a complex polymeric chemical vector. Since it is composed of humanized proteins, the likelihood of immune response is significantly diminished. The protein has a precise structure, defined by the recombinant gene from which it is expressed, which increases its chances of meeting the requirements of clinical development and regulatory approval processes [34]. Our protein vector, dsRB-SCP (dsRB-Arg_9_-ScFvJ591), is a chimeric protein that comprises: (a) a polyIC-binding moiety, consisting of two dsRNA binding domains (dsRBD) derived from human dsRNA-activated protein kinase (PKR); (b) a nine residue arginine linker (Arg_9_), to facilitate endosomal escape following endocytosis, in accordance with the findings of He et al.[35, 36]; (c) a specific targeting moiety, consisting of anti-PSMA single-chain antibody, ScFvJ591 [29, 37]. We show that dsRB-SCP successfully binds polyIC and elicits a powerful and selective killing effect on PSMA-overexpressing prostate cancer cells.

## RESULTS

### ScFvJ591 selectively targets PSMA-overexpressing prostate cancer cells and efficiently internalizes into the cells

**Figure 1:**
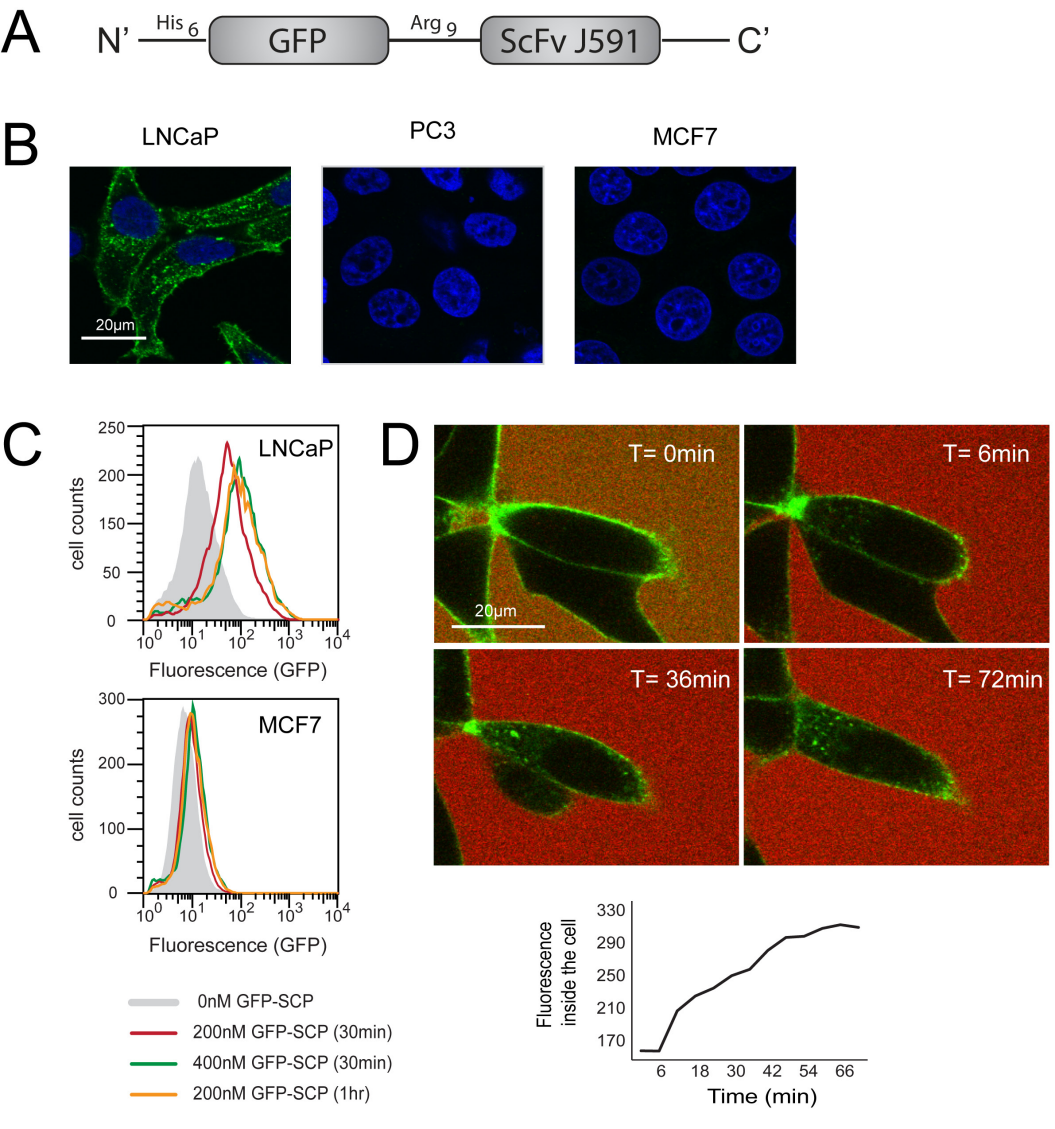
GFP-SCP binds and selectively internalizes into PSMA-overexpressing cells **A.** Schematic representation of GFP-SCP. **B.** LNCaP, PC3 and MCF7 cells were incubated with 25nM GFP-SCP for 5 h. The cells were fixed and stained with anti-GFP antibody (Cy3) and 4, 6-diamidino-2-phenylindole and viewed by laser scanning confocal microscopy. **C.** LNCaP and MCF7 cells were incubated with GFP-SCP as indicated, then subjected to flow cytometric analysis. **D.** LNCaP cells were monitored by laser confocal imaging, 0 to 72 min after the addition of 200nM GFP-SCP. Sulforhodamine-B was added to the medium immediately before adding the GFP-SCP, to mark the outside of the cells. The graph shows GFP fluorescence inside the cell, as measured using ImageJ.

We first tested whether the single chain antibody ScFvJ591 [29] could be used as a homing ligand, as part of a chimeric protein. We generated pGFP-Arg_9_-ScFvJ591, encoding GFP as a tracking marker fused to the single chain antibody against PSMA, ScFvJ591. The GFP and ScFv moieties are connected by a linker encoding an endosomal escape sequence (Figure 1A). The 56kDa recombinant protein, GFP-SCP (GFP-Arg_9_-ScFvJ591), was expressed in *E. coli* and purified in a 3-step purification process, consisting of affinity purification followed by two steps of gel filtration (Experimental Procedures).

We examined the selectivity of GFP-SCP using confocal microscopy. We incubated the chimeric protein with LNCaP cells, which overexpress PSMA, and analyzed binding after 5 hours. PC3 and MCF7 cells, which do not express PSMA, served as negative controls. The confocal images demonstrated that GFP-SCP bound to LNCaP cells and was internalized, while no binding was evident to PC3 or MCF7 cells (Figure 1B). We next compared the uptake of GFP-SCP into LNCaP and MCF7 cells using flow cytometry. The accumulation of GFP-SCP was indicated by the fluorescence shift. As expected, the observed fluorescence levels correlated with the concentration of GFP-SCP (200nM versus 400 nM) and the incubation period (30 minutes versus 60 minutes) (Figure 1C). These results suggest time-dependent and dose-dependent internalization of GFP-SCP. In contrast, in MCF7 cells, which lack PSMA, no accumulation of GFP-SCP was observed (Figure 1C).

To monitor the localization of GFP-SCP, we incubated LNCaP cells with GFP-SCP and observed them using live-cell confocal microscopy. Initially, GFP-SCP fluorescence was confined to the cell surface and no free diffusion was observed (Figure 1D). Minutes later, GFP-SCP entered the cell via endocytosis, as indicated by the appearance of small intracellular punctate structures (Figure 1D). Over time, these structures increased in number. Eventually, the fluorescence became more diffuse (Figure 1D), suggesting that the GFP might have escaped from the endosome and diffused to the cytosol. The accumulation of the GFP inside the cell increased linearly over the first 40 minutes after binding (Figure 1D). Thus, GFP-SCP was taken up quickly and selectively by PSMA-overexpressing cells.

### Production of a chimeric protein to deliver polyIC selectively to PSMA-overexpressing prostate cancer cells

**Figure 2:**
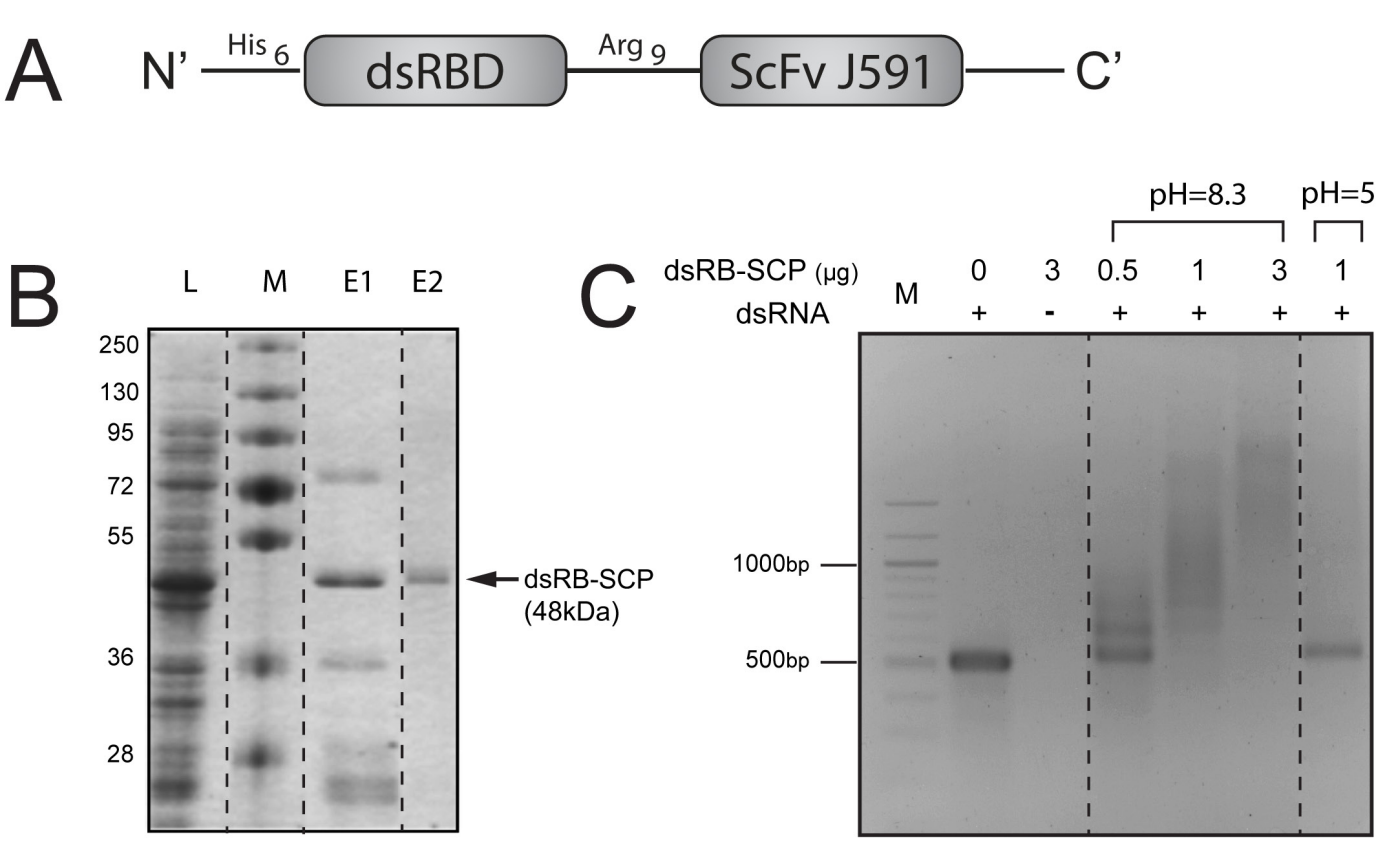
Design, expression and purification of dsRB-SCP **A.** Schematic representation of dsRB-SCP. **B.** Expression and purification of dsRB-SCP: L: Cleared lysate, M: Molecular weight marker, E1: Eluate following IMAC (nickel sepharose column), E2: Purified dsRB-SCP eluted from IEX (ion exchange column). **C.** Binding of dsRB-SCP to dsRNA. dsRB-SCP (0.5-3μg) was preincubated with 500 bp long dsRNA in binding buffer at pH 8.3 or pH 5, and electrophoresed on a 2% agarose gel. M: 100 bp DNA molecular weight marker. Dashed lines indicate where the pictures of the gels were cut and reorganized.

Once we had verified that the single chain antibody ScFvJ591 could specifically target PSMA-overexpressing cells, we designed a chimeric protein in which ScFvJ591 was fused with the two dsRNA-binding domains (dsRBD) of the human dsRNA-dependent protein kinase, PKR (Figure 2A). The 48kDa chimeric protein, dsRB-SCP (dsRB-Arg_9_-ScFvJ591), was expressed in *E. coli*, purified on nickel sepharose as a partially unfolded protein, refolded on the column, eluted and submitted to ion exchange, as described in the experimental procedures (Figure 2B).

To evaluate binding to dsRNA, dsRB-SCP was incubated with dsRNA of defined length (500 bp) and the mixture was electrophoresed on an agarose gel. The electrophoresis of dsRNA that was incubated with dsRB-SCP at physiologic pH was retarded in a dose-dependent manner, relative to the naked dsRNA control (Figure 2C), confirming that the chimeric protein bound the dsRNA. On the other hand, dsRNA that was incubated with dsRB-SCP at acidic pH migrated like naked dsRNA (Figure 2C). This indicates that the affinity of dsRB-SCP for dsRNA is low under acidic conditions, suggesting that dsRB-SCP can release its dsRNA cargo upon reaching the acidic environment of the endosome. The dsRB-SCP/polyIC complexes were visualized by cryo-transmission electron microscopy as distinct elongated structures, reminiscent of beads on a filament (Supplementary Figure 1). Similar structures were observed by Peisley et al., who looked at complexes of dsRNA and MDA5 protein [38]. These data confirm that dsRB-SCP can be used as a vector for dsRNA.

### dsRB-SCP complexed with polyIC selectively induces apoptosis in PSMA-overexpressing cells

**Figure 3:**
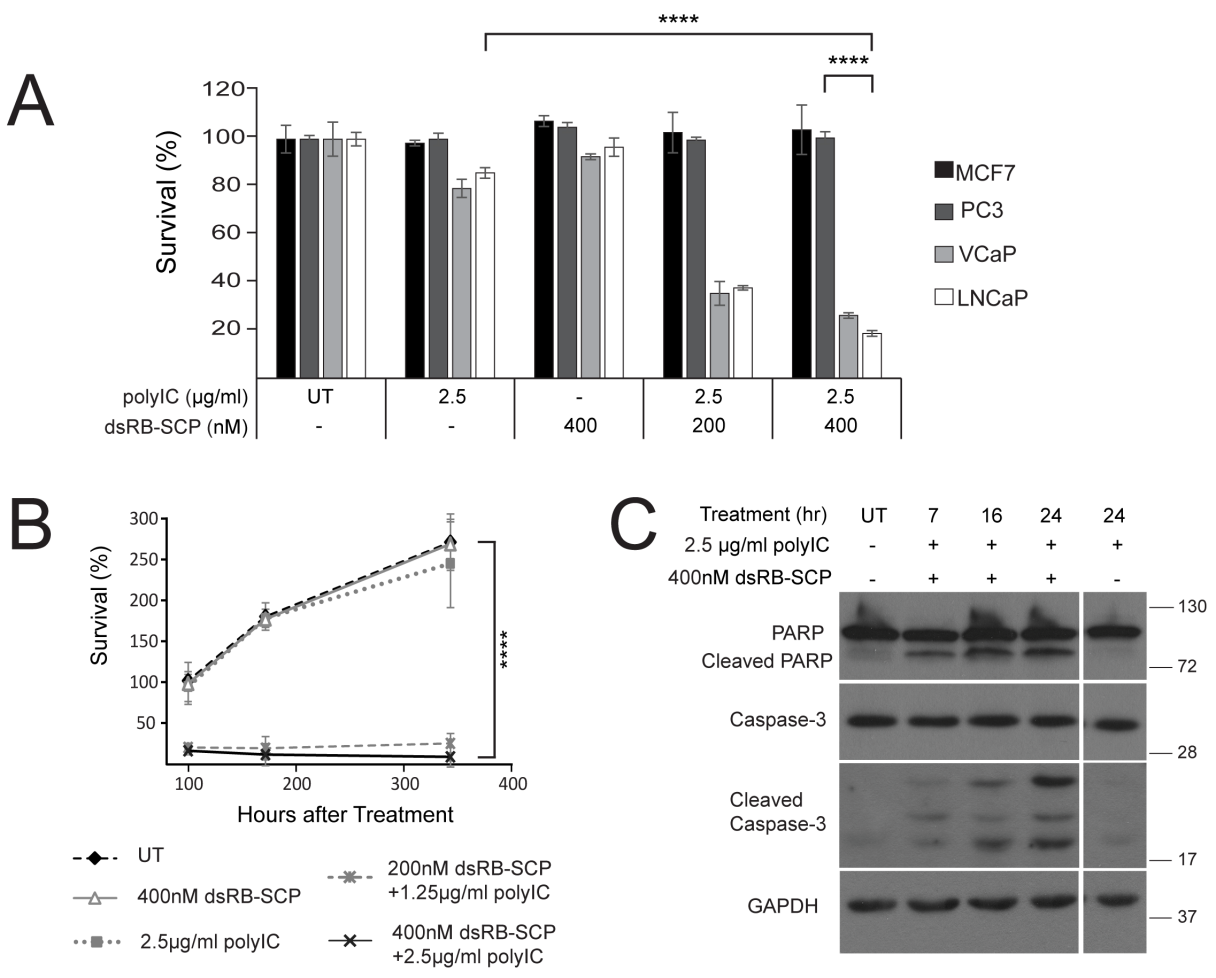
dsRB-SCP/polyIC selectively induces apoptosis of PSMA-overexpressing cells **A.** Cells were seeded in triplicate, grown overnight, and treated with dsRB-SCP/polyIC, polyIC alone or dsRB-SCP alone, as indicated, for 100 h. Viability was quantified using the CellTiter-Glo Luminescent Cell Viability Assay (Promega). Results (mean and standard deviation) are representative of two independent experiments (****P ≤ 0.0001, treatment with dsRB-SCP/polyIC of LNCaP vs PC3; ****P ≤ 0.0001 treatment of LNCaP with dsRB-SCP/polyIC vs polyIC alone). **B.** Surviving cells remained permanently arrested. Cells were seeded in triplicate, grown overnight, and treated as indicated. Medium was replaced and viability was quantified after 100/172/344 h using CellTiter-Glo (****P ≤ 0.0001 dsRB-SCP/polyIC treatment vs UT). Control cells were unable to proliferate beyond 2.5 doublings because they had reached full confluence. **C.** LNCaP cells were treated for the indicated times with dsRB-SCP/ polyIC or polyIC alone, lysed and subjected to western blot analysis to detect full-length and cleaved Caspase-3 and PARP.

We next evaluated the killing effect of the dsRB-SCP/polyIC complex using four cell lines: LNCaP and VCaP, which overexpress PSMA, and MCF7 and PC3, which do not express PSMA. Cells that did not express PSMA were not killed by the treatment (Figure 3A). dsRB-SCP selectively delivered polyIC into the PSMA-overexpressing cells (LNCaP and VCaP), killing up to 80% of the cells (Figure 3A). The remaining 20% of LNCaP cells were deemed permanently arrested, as no regrowth was observed 250 hours after washing out the chimera (350 hours after treatment) (Figure 3B). dsRB-SCP/polyIC induced cell death by activating apoptotic pathways, as indicated by the cleavage of caspase-3 and PARP (Figure 3C). In cells treated with polyIC alone, no cleavage of caspase-3 or of PARP was detected (Figure 3C). These data show that dsRB-SCP delivers polyIC selectively into PSMA-overexpressing cells, leading to their apoptosis.

### dsRB-SCP/polyIC treatment induces cytokine secretion and chemotaxis of immune cells

**Figure 4:**
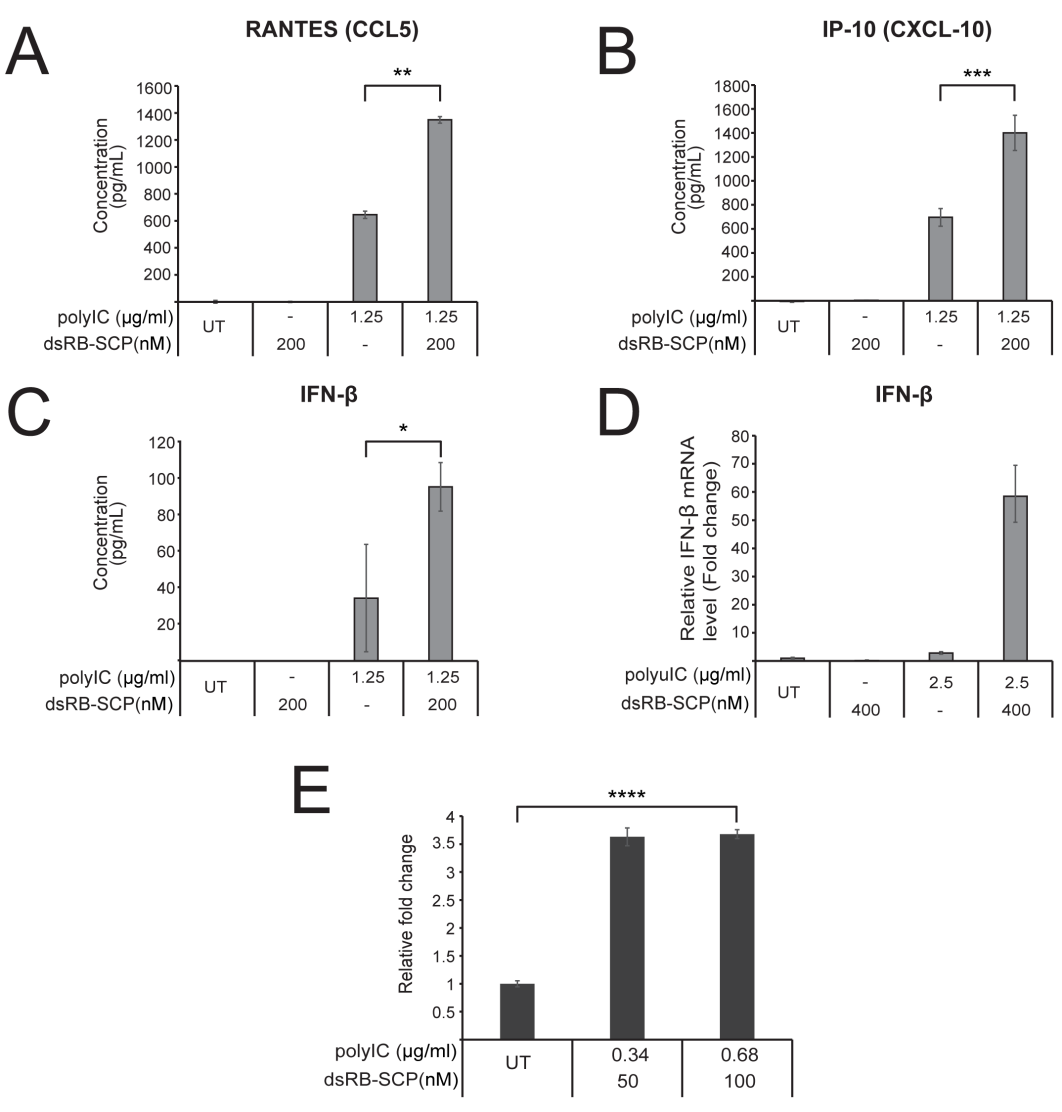
dsRB-SCP/polyIC leads to the secretion of pro-inflammatory cytokines and the recruitment of PBMCs **A.-C.** LNCaP cells were treated as indicated for 48 h, after which medium was collected. IP-10, RANTES and IFN-β in the medium were measured by ELISA assays. **D.** IFN-β transcription was measured by qRT-PCR of LNCaP cells treated as indicated for 4 h. Values are IFN-β mRNA levels, normalized to GAPDH mRNA and expressed as fold change compared to untreated (UT) cells. All assays were conducted in triplicate and data are presented as mean concentration with standard deviation (***P ≤ 0.001, **P ≤ 0.01, *P ≤ 0.05, dsRB-SCP/polyIC treatment vs polyIC alone). Results are representative of two independent experiments. **E**.. dsRB-SCP/ polyIC induces chemotaxis of PBMCs. LNCaP cells were grown and treated as indicated. 48 h after treatment, the cell medium was transferred to the lower chamber of a Transwell chemotaxis plate. PBMCs were added to the upper chamber, and the plates were incubated for 3.5 h. Then, medium was collected from the lower chamber, in order to quantify lymphocytes that had migrated to the lower chamber. Lymphocytes in the collected medium were quantified by FACS. The number of lymphocytes that had migrated in the treated group is shown as mean fold change with standard deviation, relative to the number of cells that had migrated in the untreated group (****P ≤ 0.0001, dsRB-SCP/polyIC treatment vs untreated (UT)).

The presence of dsRNA inside the cell activates the production of anti-proliferative and pro-apoptotic cytokines and chemokines [39]. To determine whether dsRB-SCP/polyIC can trigger similar effects we analyzed the production by LNCaP cells of three major cytokines: IP-10 and RANTES, which are both involved in the chemo-attraction of immune cells, and IFN-β, which plays a key role in the differentiation of immune cells [40]. The secretion of IP-10, RANTES and IFN-β into the medium, as measured by ELISA, was partially induced by polyIC alone, as reported previously [41]. Treatment with dsRB-SCP/polyIC led to a further 2-fold increase in the secretion of all three cytokines (Figure 4A-4C). Similarly, IFN-β expression was not affected by polyIC or dsRB-SCP alone, but treatment with dsRB-SCP/polyIC led to very strong induction of IFN-β expression, as measured by qRT-PCR (Figure 4D).

To study whether the secreted cytokines attract immune cells, we examined whether the medium from dsRB-SCP/polyIC-treated LNCaP cells induced the chemotaxis of freshly isolated PBMCs. Almost four times as many PBMCs migrated towards conditioned medium from dsRB-SCP/polyIC-treated cells as towards medium from untreated cells (Figure 4E). Thus, targeted delivery of polyIC into PSMA-overexpressing cells leads to cytokine secretion and consequent immune cell chemo-attraction.

### Powerful bystander effects induced by dsRB-SCP/polyIC

**Figure 5:**
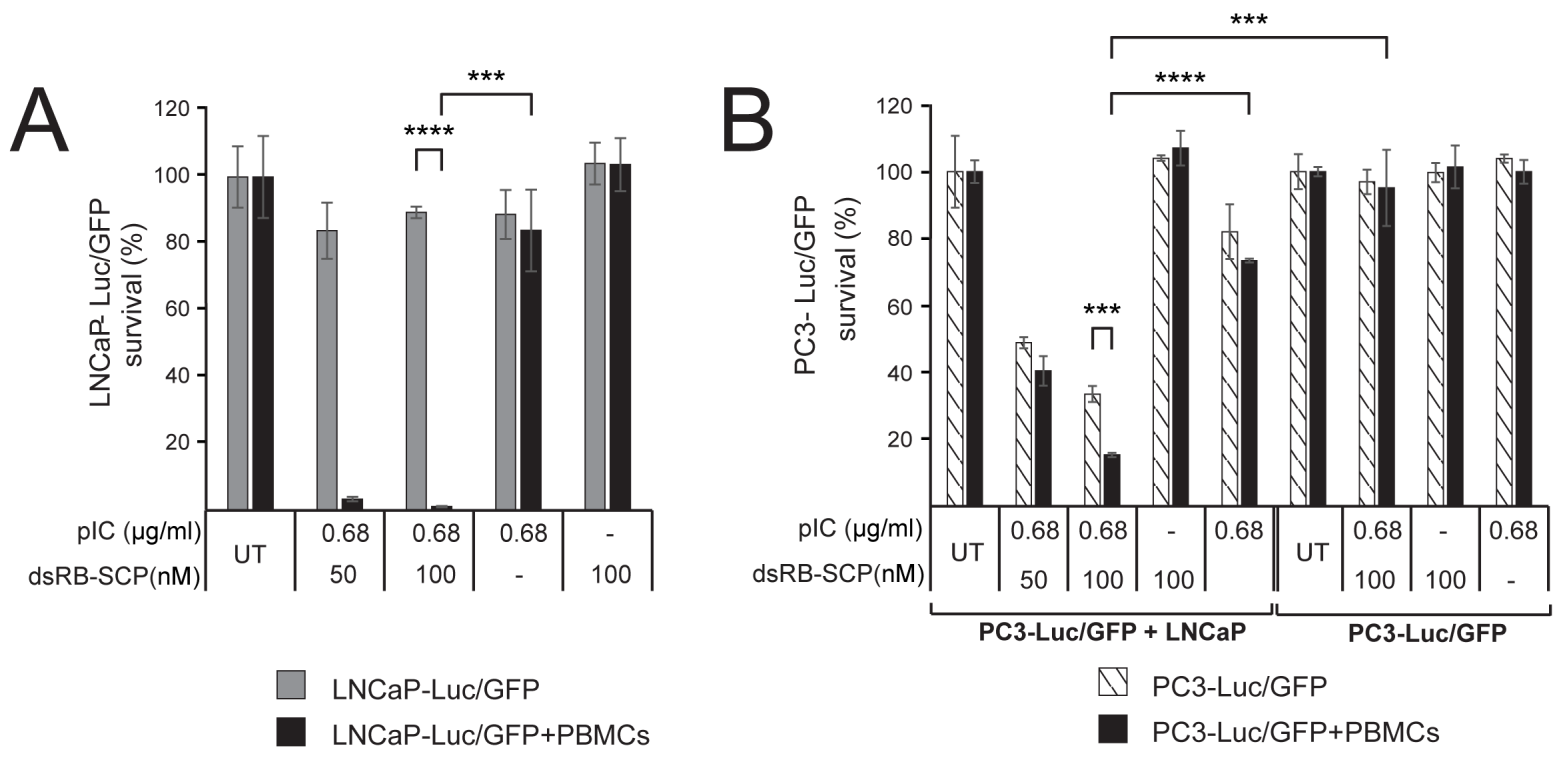
dsRB-SCP/polyIC induces direct and PBMC-mediated bystander effects **A.** LNCaP-Luc/GFP cells were untreated or treated with dsRB-SCP/polyIC, polyIC alone or dsRB-SCP alone, as indicated. After 24 h, PBMCs were added to the test wells (black bars), and medium without PBMCs was added to the control wells (gray bars). The survival of LNCaP-Luc/GFP cells was measured using the Luciferase Assay System (Promega) (****P ≤ 0.0001, LNCaP-Luc/GFP+PBMCs vs LNCaP-Luc/GFP, ***P ≤ 0.001 dsRB-SCP/polyIC treatment vs polyIC alone). **B.** PC3-Luc/GFP cells co-cultured with PBMCs, in the presence or absence of LNCaP cells. PC3-Luc/GFP+LNCaP: LNCaP cells were untreated or treated with dsRB-SCP/polyIC, dsRB-SCP alone or polyIC alone, as indicated. After 24 h, PC3-Luc/GFP cells were added to the culture. 6 h later, PBMCs (black bars) or medium (hatched bars) was added to the culture. PC3-Luc/GFP: LNCaP growth medium, in the absence of cells, was untreated or treated with dsRB-SCP/polyIC, dsRB-SCP alone or polyIC alone, as indicated. After 24 h, PC3-Luc/GFP cells were added, and 6 h later either PBMCs (black bars) or medium (hatched bars) was added. The survival of PC3-Luc/GFP cells was measured using the Luciferase Assay System (Promega) (***P ≤ 0.001, PC3-Luc/GFP+LNCaP+PBMCs vs PC3-Luc/GFP+LNCaP; ****P ≤ 0.0001 PC3-Luc/GFP+LNCaP+PBMCs, dsRB-SCP/polyIC treatment vs polyIC alone; ***P ≤ 0.001, PC3-Luc/GFP+LNCaP+PBMCs vs PC3-Luc/GFP+PBMCs). Each experiment was conducted in triplicate. Representative data (mean and standard deviation) from three independent experiments are shown.

We next tested whether the recruited immune cells could evoke an immune-cell-mediated bystander effect. We treated LNCaP-Luc/GFP cells, which stably express luciferase, with a low dose of dsRB-SCP/polyIC, followed by co-incubation with PBMCs. Luciferase activity served to measure the viability of the LNCaP-Luc/GFP cells. At the low doses used here, luciferase activity was barely affected when PBMCs were absent. In contrast, when PBMCs were added, the LNCaP-Luc/GFP cells were eradicated (Figure 5A). These results suggest that dsRB-SCP/polyIC induces a powerful bystander effect, mediated by immune cells among the PBMCs.

To evaluate whether dsRB-SCP/polyIC also induces a direct bystander effect, LNCaP cells were co-incubated with PC3-Luc/GFP cells, which do not express PSMA. dsRB-SCP/polyIC treatment resulted in the killing of up to 60% of the PC3-Luc/GFP cells (Figure 5B). Since PC3-Luc/GFP cells are not targeted by dsRB-SCP/polyIC (Figure 5B), we infer that the death of these cells is the result of a direct bystander effect elicited by the dsRB-SCP/polyIC-targeted LNCaP cells. The addition of human PBMCs to this co-culture system led to a significant increase in the killing rate of the PC3-Luc/GFP cells (Figure 5B), indicating that the direct and indirect bystander effects both operate under these conditions.

### dsRB-SCP/polyIC destroys tumor spheroids

**Figure 6:**
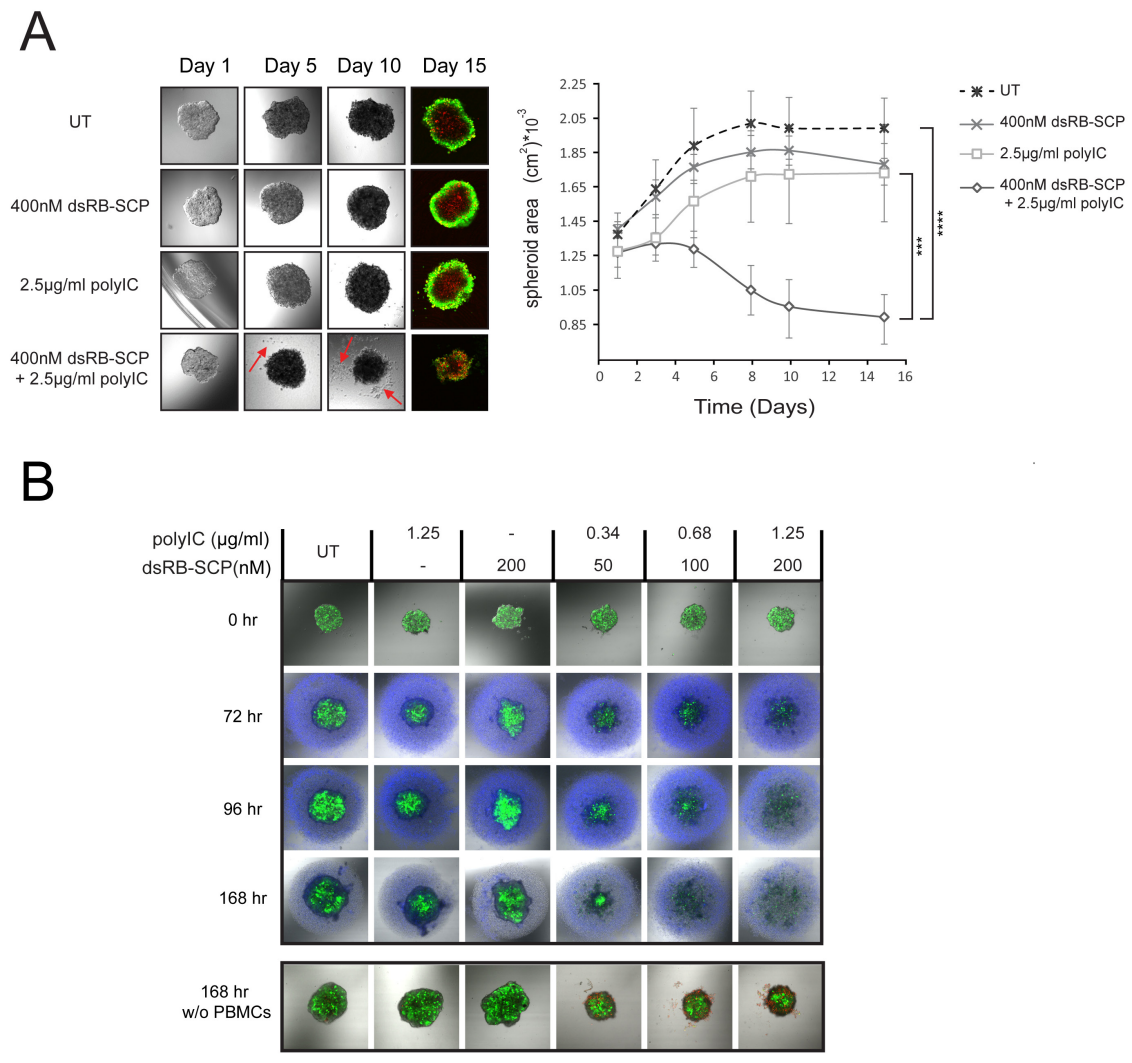
dsRB-SCP/polyIC treatment together with PBMCs leads to the destruction of LNCaP spheroids **A.** Spheroids of 300-400μm diameter were divided into four treatment groups (*n* = 4): untreated (UT), dsRB-SCP alone, polyIC alone and dsRB-SCP/polyIC. The spheroids were treated four times, on days 1, 2, 4 and 5, and then cultured for 10 additional days. Spheroid images were captured by laser scanning confocal microscopy at the indicated times; a series of 4 snapshots of a representative spheroid from each treatment group is shown. Note the prominent shedding of cells from the treated spheroid (red arrows). On Day 15, spheroids were labeled with Calcein AM (living cells; green) and Propidium Iodide (PI; dead cells; red). Areas of spheroids, measured using ImageJ, are shown in the graph (mean and standard deviation, ****P ≤ 0.0001 dsRB-SCP/polyIC treatment vs untreated cells, ***P ≤ 0.001 dsRB-SCP/polyIC vs dsRB-SCP alone or polyIC alone). **B**. Upper panel: LNCaP-Luc/GFP spheroids of 300-400μm diameter were divided into six treatment groups, as indicated (*n* = 4). After 24 h, 8×10^4^ PBMCs labeled with CellTracker™ Violet BMQC (Invitrogen) were added to the spheroids. A series of snapshots of one representative spheroid per treatment is presented. Lower panel: PBMC medium without cells was added to the spheroids. Spheroids in both panels were captured by laser scanning confocal microscopy 0, 72, 96, 168 h after treatment initiation. Living cells were detected by their GFP fluorescence. Spheroid area was not quantified as the structure became totally diffuse shortly after the treatment began. PI was added to the spheroids in the lower panel, to highlight the dead cells. PI staining of upper panel is not shown, as there is no way to distinguish between dead LNCaP-Luc/GFP cells and dead PBMCs. The complete time course of the lower panel is shown in Supplementary Figure 2.

*In vitro* 3D models display some elements of the architecture of human tumors [42] and feature high resistance to anti-cancer drugs [43]. We therefore evaluated the efficacy of dsRB-SCP/polyIC in a 3D tumor spheroid model. LNCaP spheroids were generated and allowed to reach a diameter of 300-400 μm. The spheroids were then transferred to polyHEMA-coated plates and treated repeatedly with dsRB-SCP/polyIC (400 nM dsRB-SCP, 2.5 μg/ml polyIC) over the course of 5 days. By day 5, the spheroids that were treated with dsRB-SCP/polyIC began to shrink and shed dead cells, while the untreated spheroids continued to grow (Figure 6A). On day 15, the spheroids were stained with calcein AM and propidium iodide to monitor viability (Figure 6A). The dsRB-SCP/polyIC-treated spheroids demonstrated significant structural damage and contained large numbers of dead cells (Figure 6A). In contrast, the untreated spheroids and spheroids treated with only polyIC or only dsRB-SCP, maintained a typical intact structure [23], where the cells at the surface were alive and the cells at the core were necrotic, as indicated by calcein-AM versus propidium iodide staining. (Figure 6A).

To test the immune-cell-mediated bystander effect on the spheroids, we added PBMCs to treated spheroids. LNCaP-Luc/GFP spheroids were treated once with dsRB-SCP/polyIC, and 24 hours later freshly isolated PBMCs were added to the culture. Even at the lowest dose of dsRB-SCP/polyIC, spheroid disassembly was already evident 72 hours after the initiation of dsRB-SCP/polyIC treatment (48 hours after PBMC addition) (Figure 6B upper panel). At higher doses, complete spheroid destruction was observed 96 hours after the initiation of the treatment. 72 hours later only dead cells, which showed no GFP fluorescence, were evident (Figure 6B upper panel). In the absence of PBMCs (Supplementary Figure 2), dsRB-SCP/polyIC treatment resulted in visible cell death and disassembly of the spheroid but the effect was weaker than in the presence of PBMCs (Figure 6B lower panel shows endpoint at 168 hours). Thus, dsRB-SCP/polyIC has a potent effect on spheroids, and this effect is greatly magnified by the addition of immune cells.

## DISCUSSION

There are currently no effective clinically approved treatment options for CRPC patients [2]. While PSMA is the target of choice for targeted treatment of prostate cancer in general and CRPC tumors in particular [44], no PSMA-targeted drugs have entered the clinic to date. Our therapy utilizes PSMA to facilitate the targeted delivery of polyIC into prostate cancer cells. PolyIC is a well-known anti-tumor immune adjuvant. However, its therapeutic window is limited due to its strong toxic side effects when delivered systemically [45]. By targeting polyIC to the tumor, systemic toxicity can be avoided [14]. Our strategy uses polyIC not only to directly kill cells that overexpress PSMA, but also to activate direct and immune-cell-mediated bystander effects, in order to eradicate heterogeneous tumors.

Our previous studies focused on the use of chemical vectors to target polyIC to EGFR- and HER2-overexpressing cancer cells [14, 33]. Proteins have several potential advantages as therapeutics over chemical compounds, as proteins function in a highly specific manner and feature consistent structures and low immunogenicity [34, 46]. These advantages, as well as recent technical advancements in the field of protein engineering [47], motivated us to develop protein-based drugs. Here, we generated a novel chimeric protein, dsRB-SCP, which can selectively deliver polyIC into PSMA-overexpressing cancer cells.

ScFvJ591 [29], the targeting moiety of our chimeric protein, was derived from the monoclonal antibody (mAb) J591, which recognizes the extracellular domain of PSMA [48]. mAb J591 has been used successfully in clinical studies. As an imaging tracer, mAb J591 linked to radionuclides ^89^Zr or ^111^I gave excellent results in the detection of bone lesions [49, 50]. mAb J591 linked to ^177^Lu was used in radio-immunotherapy to successfully target circulating tumor cells [20, 31]. mAb J591 was used to deliver the cytotoxic drug maytansinoid-1 (DM1) in a phase I clinical trial [21]. In all clinical applications, mAb J591 exhibited low immunogenicity. We chose to use the single-chain version of the full antibody, ScFvJ591, which consists of the variable regions of the heavy (VH) and light (VL) chains of mAb J591, joined together by a flexible peptide linker [29]. This short single-chain version is easier to incorporate than the full antibody into an engineered chimeric protein expressed in bacteria [51]. Our GFP-SCP chimera entered PSMA-overexpressing cells rapidly and selectively, showing that both the single chain ScFvJ591 and the GFP were expressed in active conformations in the context of the recombinant fusion protein. The high specificity and affinity of the ScFvJ591-containing chimeric protein are crucial for a potential therapy.

To construct a chimeric protein that would specifically bind and carry polyIC, we utilized the dsRNA binding domains (dsRBD1 and dsRBD2) isolated from the dsRNA-activated protein kinase (PKR). Previous studies showed that PKR has a high affinity for polyIC [52–54] and that isolated dsRBDs can facilitate the delivery of siRNA into cells [55]. Based on these reports we recently used the isolated dsRBDs to deliver polyIC to EGF-targeted cells successfully [56].

The expression and purification of a correctly folded, active chimeric protein is challenging. Here, we had the additional complication that the dsRB domain binds host nucleic acids [57]. We designed a purification process incorporating an unfolding and re-folding protocol, which successfully released the host nucleic acids [56]. We proved that the dsRB domain was functional using a gel mobility shift assay [58] (Figure 2C). Our purification process successfully generated purified dsRB-SCP with low batch-to-batch variations.

The immunostimulatory effect of polyIC is mainly mediated through the Toll-like Receptor3 (TLR3), which is part of the antiviral defense system. TLR3 is expressed on endosomal membranes and recognizes polyIC extracellularly [59]. Upon internalization, polyIC can escape from the endosomes into the cell cytosol, where it activates additional antiviral defense systems, including PKR, Retinoic acid-Inducible Gene I (RIG-1) and Melanoma Differentiation-Associated gene 5 (MDA5) [60]. To facilitate endosomal escape we inserted a peptide linker consisting of nine arginine residues, between the targeting and the polyIC-binding moieties. We chose the Arg_9_ peptide for two reasons. First, it is a synthetic derivative of HIV-I Tat protein, which is widely used as a membrane penetrating peptide. The peptide's high positive charge creates strong electrostatic interactions with the endosomal membrane, leading to its puncture [35, 36, 61]. Second, the Arg_9_ peptide contains a sequence that can be cleaved by furin, an endoprotease that is localized in the endosome [62]. The Arg_9_ sequence has been used to enable endosomal escape in the development of recombinant immunotoxins, with good results [35, 63].

dsRB-SCP was designed to bind polyIC via the dsRBD moiety, target prostate cancer cells using the ScFvJ591 moiety, and then release the polyIC cargo inside the endosome. To verify that the particles were small enough to enter the endosome (less than 200 nm, according to Rejman et al. [64]), the complex was examined using cryo-transmission electron microscopy (Supplementary Figure 1). The acidic environment of the endosome influences the electrostatic interactions between the charged residues in the binding domain, as was shown with other dsRNA binding proteins[65]. dsRB-SCP achieved high killing efficiency using low doses of polyIC (Figure 3A), which were ineffective in the absence of dsRB-SCP (Figure 3A). Paone et al. succeeded in triggering apoptosis in LNCaP cells using 10-fold higher doses of naked PolyIC [66]. dsRB-SCP/polyIC triggered apoptosis specifically in PSMA-overexpressing cells within hours after internalization (Figure 3), fulfilling the criteria for effective therapy of selective and rapid action.

In addition to launching a strong direct attack on the targeted PSMA-overexpressing cancer cells, the strategy described here invokes a holistic approach to targeted therapy. Unlike kinase inhibitors, for example, which inhibit one or a small number of specific cancer driver molecules [67, 68], our therapy induces bystander effects that: (1) activate multiple killing pathways at the same time, thus preventing targeted cells from developing resistance and, (2) kill neighboring tumor cell sub-populations, overcoming the heterogeneous nature of tumors. These pathways activate the secretion of toxic cytokines (inducing a direct bystander effect) and the adaptive immune response (inducing an indirect, immune-cell-mediated bystander effect) [60]. The bystander effects are mediated by the induction of cytokines such as IFN-β (Figure 4C-4D), and chemokines such as IP-10 and RANTES (Figure 4A-4B), known to be responsible for the attraction of monocytes and T lymphocytes [69, 70]. Indeed, medium from the treated cells successfully supported chemo-attraction of PBMCs (Figure 4E). dsRB-SCP successfully activated direct and immune-cell-mediated bystander effects, amplifying the killing of PSMA-overexpressing cells and extending the effect to neighboring PSMA-negative cancer cells (Figure 5). These *in vitro* findings suggest that the treatment should be effective against heterogeneous tumors, in vivo.

To better predict the clinical efficacy of dsRB-SCP/polyIC we used a tumor spheroid model. Cancer cells cultured as monolayers often show misleading sensitivity to therapeutic agents. Tumors feature cell-cell adhesion and three-dimensional shape, leading to “multi-cellular resistance” (MCR), which cannot be replicated in two-dimensional cultures [71]. Tumor cells cultured as spheroids present a multi-layer cell system that has many features of the three-dimensional tumor and therefore has greater potential to predict clinical efficacy [43, 72, 73]. Treatment of tumor spheroids with dsRB-SCP/polyIC resulted in visible structural damage (loss of integrity), probably due to the loss of adhesion between dead cells (Figure 6A). Adding immune cells to the spheroid model greatly amplified the response and accelerated the tumor disintegration time (Figure 6B). In both monolayer and spheroid cultures, the bystander effects were activated at extremely low doses of polyIC, suggesting that extensive tumor killing can be obtained *in vivo*, without toxic side effects.

In conclusion, we present a potent targeted therapy for metastatic prostate cancer, which leverages the antitumor effect of polyIC and delivers it specifically to cancer cells, using a novel chimeric recombinant protein vector. Our treatment demonstrates high effectiveness. It eradicates targeted and neighboring untargeted cancer cells in monolayer cultures, and rapidly and efficiently destroys tumor spheroids. It does so by activating apoptotic cancer cell death, as well as powerful direct and immune-cell-mediated bystander effects. Our treatment has two major advantages in the clinical setting. First, it can be used with low doses of polyIC, especially in patients who are not immune compromised, thus minimizing polyIC toxicity [74]. Second, tumors are heterogeneous, with only some cells overexpressing PSMA. dsRB-SCP overcomes this challenge and holds promise of substantial efficacy, because of its ability to harness the immune system against the whole tumor and not only against the targeted sub-populations.

## EXPERIMENTAL PROCEDURES

### Cloning of GFP-SCP and dsRB-SCP

Plasmids pGFP-SCP (encoding GFP linked via Arg_9_ to the single chain antibody ScFvJ591 against PSMA; 56 kDa; Figure 1A) and pdsRB-SCP (encoding dsRB of human PKR linked via Arg_9_ to ScFvJ591; 48 kDa; Figure 2A) were constructed as follows: SCP (single chain antibody against PSMA, ScFvJ591) was amplified by PCR from plasmid SFG-Pz1 [29], using primers SCP-N and SCP-C. GFP was amplified by PCR from plasmid pEGFP-N3 (Clontech), using primers GFP-N and GFP-C. dsRB was amplified by PCR from plasmid DRBM-DT-EGF [56] using primers dsRB-N and dsRB-C. To prepare the Arg_9_ linker (GSRRRRRRRRGRKA), oligonucleotide 9ARG1 was annealed to its complementary oligonucleotide 9ARG2. The oligonucleotides used are listed in Table 1. GFP-SCP was constructed in several stages in the bacterial expression vector pET28a (Novagen): GFP was cloned after the His_6_ tag of plasmid pET28a, between the *Nde*I and *Bam*HI restriction sites, SCP was cloned between the *Hin*dIII and *Xho*I sites, and the Arg_9_ linker was inserted between the *Bam*H1 and *Hin*dIII sites, to give the fusion His_6_-GFP-Arg_9_-SCP (Figure 1A and Supplementary Data). For the construction of dsRB-SCP, the GFP fragment was replaced with the dsRB sequence using restriction sites *Nde*I and *Bam*HI, to give the fusion His_6_-dsRB-Arg_9_-SCP (Figure 2A and Supplementary Data). The constructs were sequenced at The Center for Genomic Technologies at The Hebrew University of Jerusalem.

**Table 1:** Oligonucleotides used for the construction of pGFP-SCP and dsRB-SCP

Name	Sequence 5′ to 3′
SCP-N	TTTACTCGAGCGGAGGTGCAGCTGCAGC
SCP-C	TTTTGCTCAGCGCCGTTACAGGTCC AGCCATG
GFP-N	TTTTCATATGGTGAGCAAGGGCG
GFP-C	TAAGGATCCGCCACCGCCGCTTTT CTTGTACAGC
dsRB-N	TTTCATATGATGGCTGGTGATC
dsRB-C	TTAGGATCCGCCACCGCCGCTCTCCGATAAGATC TGCAG
9ARG1	GATCCCGTCGTCGCCGTCGTCGCCGTCGCGGCCGCAA
9ARG2	AGCTTTGCGGCCGCGACGGCGACGACGGCGACGACGG

### Expression of GFP-SCP and dsRB-SCP

The chimeric proteins were expressed in *E. coli* BL21*trxB*(DE3) (Novagen, Madison, WI, USA) which had been transformed with plasmid pRARE, which encodes tRNAs for rare codons. The bacteria were grown at 37°C, in 2XYT medium, supplemented with 25 μg/ml chloramphenicol, 30 μg/ml kanamycin, 100 μg/ml ampicillin, 1% glucose and 5% NPS buffer (1M KH_2_PO_4_, 1M Na_2_HPO_4_, 0.5M (NH_4_)_2_SO_4_). When the culture reached OD_600_ ∼ 0.3, 0.1% glycerol and 0.1mM L-glutamic acid were added, and the culture was moved to 42 °C, to induce the expression of *E. coli* chaperones and enhance protein solubility. When the culture reached O.D_600_∼0.9, it was cooled down on ice and transferred to 14 °C. After a 10 minute adjustment period, IPTG was added to 0.5 mmol/L, followed by incubation for 24 h at 14 °C. The bacteria were harvested and the pellet stored at -80°C until purification.

### Purification of GFP-SCP and dsRB-SCP

GFP-SCP: The pellet obtained from 1.2 L of *E.coli* BL21*trxB*(DE3, pRARE, pGFP-SCP) was thawed on ice in 60ml binding buffer (Buffer A, 30mM HEPES pH 8.3, 0.5M NaCl, 10% glycerol, 10mM imidazole) supplemented with a protease inhibitor cocktail, 3mg/ml lysozyme and DNase, and lysed using a LV1 microfluidizer (Microfluidics, Newton, MA, USA). The extract was clarified by centrifugation for 30 minutes (15,000×g, 4°C), loaded onto an 8 ml nickel sepharose FF IMAC column (GE Healthcare, Basel, Switzerland), and washed with 10 column volumes (CV) of binding buffer, followed by 6 CV of 5% Buffer B (30mM HEPES pH 8.3, 0.5M NaCl, 10% glycerol, 1M imidazole), 6 CV of 10% Buffer B and 1 CV of 15% Buffer B. The protein was eluted with 60% Buffer B. Fractions containing the chimera (8 ml total) were loaded onto a 500 ml Sephacryl S-200 gel filtration column (GE Healthcare) pre-equilibrated with GF buffer (30 mM HEPES pH 8.3, 0.5 M NaCl, 10% glycerol). The fractions eluted after 0.5 CV were pooled, concentrated using Vivaspin-20 (MWCO: 30000, GE Healthcare) and loaded onto 350 ml Superdex-75. The fractions eluted after 0.5 CV were subjected to SDS–PAGE and stained with InstantBlue (Expedeon, Harston, UK). The fractions that contained highly purified chimera were pooled, concentrated using Vivaspin-20, and stored in aliquots at -80°C.

dsRB-SCP: The pellet obtained from 6 L of *E.coli* BL21*trxB*(DE3, pRARE, pdsRB-SCP) was thawed in 300 ml binding buffer A supplemented with protease inhibitors, lysozyme and DNase, lysed and clarified as above. To release bound host nucleic acids, the cleared lysate was mixed 1:1 (vol:vol) with 8 M urea. The mixture was incubated at 4 °C for 1.5 hours and then loaded onto 60 ml nickel sepharose FF column pre-equilibrated with buffer C (Buffer A supplemented with 0.5% Tween 80 and 4M urea), and washed with 12.4 CV Buffer C. To refold the protein, a linear gradient of Buffer C to Buffer D (Buffer A supplemented with 0.5% Tween 80), 10 CV, 0.8 ml/min flow was applied. The column was washed with 3 CV of 10% and 3 CV of 25% Buffer E (30 mM HEPES pH 8.3, 0.5 M NaCl, 10% glycerol, 500 mM imidazole, 0.5% Tween 80), and the protein was eluted with 100% buffer E. The fractions containing the chimera were pooled and diluted 1:1 with dilution buffer (30 mM MES pH, 10% Glycerol, 0.5% Tween). The diluted protein was clarified by centrifugation for 30 minutes (15,000×g, 4°C) and loaded onto a 66 ml Fracto-gel EMD SO3 IEX column (Merck, Darmstadt, Germany). A manual step gradient (7 CV) of Buffer F (30 mM MES pH, 100 mM NaCl, 10% Glycerol, 0.001% Tween) and 25%, 27%, 30%, 37% and 38% Buffer G (30 mM HEPES pH 8.3, 2 M NaCl, 10% glycerol, 0.001% Tween 80) was applied. Samples of the eluted fractions were subjected to SDS–PAGE and stained with InstantBlue. Fractions that contained purified chimera were pooled, concentrated, and stored at -80°C as above. Judging from the InstantBlue stain (Figure 2B), the chimera was >95% pure. The yield was 3.8 mg protein from 6 L culture.

Cell lines LNCaP cells were cultured in RPMI 1640 medium supplemented with 10 mM HEPES pH 7.4 and 1 mM sodium pyruvate. VCaP cells were cultured in DMEM (Dulbecco's Modified Eagle Medium). PC3 and DU145 cells were cultured in MEM (Minimum Essential Medium) supplemented with 1% non-essential amino acids, 1 mM sodium pyruvate, 10 mM Hepes pH 7.4 and 1% MEM vitamin mixture. MCF7 cells were cultured in RPMI 1640 medium. All tissue culture media were supplemented with penicillin (100 U/ml), streptomycin (100 mg/l) and 10% FBS (fetal bovine serum). All cell lines were purchased from the American Type Culture Collection (ATCC, Rockville, MD, USA), tested and shown to be mycoplasma-free. LNCaP-Luc/GFP and PC3-Luc/GFP were generated using lentiviral vectors encoding the fusion gene luciferase-GFP (Luc/GFP) as previously described [33]. PBMCs were isolated from fresh human peripheral blood by standard Ficoll density-gradient centrifugation [14]. All cells were incubated at 37 °C with 5% CO_2_ in a humidified incubator. All cell culture reagents were purchased from Biological Industries, Bet Ha’emek, Israel.

### Flow cytometry

Cells were plated onto 12-well plates at a density of 10^5^ cells per well, grown for 40 hours and incubated with GFP-SCP at 37°C. After incubation, the cells were trypsinized, washed in PBS, re-suspended in 1 ml cold PBS and subjected to flow cytometry analysis using BD FACS ARIAIII (BD Biosciences, USA) equipped with a 488 nm laser. 10,000 cells were acquired for each treatment. The cells were gated to include only live cells and the subpopulation was analyzed for GFP levels. All data were analyzed using FlowJo software (Becton Dickinson, Franklin Lakes, NJ, USA).

### Immunocytochemistry

LNCaP, PC3 and MCF7 cells were grown for 48 hours and incubated with 25nM GFP-SCP for 5 hours at 37°C. After incubation cells were fixed with 4% paraformaldehyde, washed twice with PBS, permeabilized and stained with goat anti-GFP antibody (1:1000, ab5450, Abcam, Cambridge, MA, USA ), followed by incubation with DyLight 488-conjugated anti-goat secondary antibody (1:300, Jackson ImmunoResearch Laboratories, West Grove, PA, USA). 4, 6-diamidino-2-phenylindole (DAPI) was used to stain DNA. Stained samples were observed with a confocal microscope, FLUOVIEW FV-1000 (Olympus, Tokyo, Japan).

### Live cell imaging

GFP-SCP localization was observed in live LNCaP cells, using time-lapse confocal microscopy (FLUOVIEW FV-1000). LNCaP cells were grown for 48 hours in 8-well μ-slides (Ibidi, GmbH, Munich, Germany). After changing the medium, 200 nM GFP-SCP was added directly to the chamber, the cells were immediately observed and subsequent images were taken every 6 minutes, for 72 minutes. The cells were incubated at 37°C with 5% CO_2_, throughout the experiment. The images were analyzed using FLUOVIEW Viewer software (Ver.4.2).

### dsRNA Electrophoretic Mobility Shift Assay (EMSA)

500 bp long dsRNA transcribed from the control template of the MEGAscript RNAi Kit (Ambion, Austin, TX, USA) was used to evaluate the binding of the purified protein to dsRNA. 1 μg of dsRNA was incubated for 30 minutes with increasing amounts of purified dsRB-SCP (0.5-3μg) in binding buffer (30 mM HEPES pH 8.3, 0.5 M NaCl, 10% glycerol) or in acidic buffer (0.5 M K-acetate pH5, 10% glycerol), and the mixture was electrophoresed on a 2% agarose gel. The gel was visualized by staining with ethidium bromide.

### Preparation of dsRB-SCP/polyIC complex

Low molecular weight (LMW) polyIC (InvivoGen, Toulouse, France) was used for all of the experiments. To prepare dsRB-SCP/polyIC, dsRB was pre-incubated with polyIC at the concentrations indicated in the text in binding buffer (30 mM HEPES pH 8.3, 0.5 M NaCl, 10% glycerol), for 45 minutes at room temperature, before addition to the cells. For controls, PolyIC and dsRB-SCP were each pre-incubated alone for 45 minutes at room temperature in binding buffer.

Complexes of dsRB-SCP/polyIC, polyIC alone and dsRB-SCP alone, prepared as above, were dialyzed for 1.5 hours against 30mM HEPES pH8.3, 0.5M NaCl to remove the glycerol, using Slide-A-Lyzer MINI dialysis devices, 10K MWCO (Thermo Fisher Scientific Inc., MA, USA). For direct imaging using cryo-transmission electron microscopy (cryo-TEM), a drop (3μl) of sample was placed on a glow discharged TEM grid (300-mesh Cu grid) pre-coated with a holey carbon film (Lacey substrate, Ted Pella Ltd., Redding CA, USA). To obtain a vitrified thin film, excess sample was drained using filter paper and the grid was immediately plunged into liquid ethane pre-cooled by liquid nitrogen, using a Vitrobot Mark IV (FEI Company, Hillsboro, OR, USA). Using a cold stage unit (Gatan model 626), the vitrified films were transferred to the electron microscope (FEI Tecnai 12 G2 TWIN, FEI Company, Hillsboro, OR, USA) and examined at -177 °C. The images were recorded with a 4k x 4k FEI Eagle CCD camera in low dose mode and analyzed using Fiji image processing software.

### Survival assay

LNCaP, VCaP, PC3 and MCF7 cells were seeded in 96-well plates in triplicate (5000 cells/well) and grown overnight. dsRB-SCP/polyIC, polyIC alone or dsRB-SCP was added to the cells, which were then incubated for another 100 hours. Survival was measured using the CellTiter-Glo Luminescent Cell Viability Assay (Promega, Madison, WI, USA). To test whether the ∼20% of cells remaining after treatment were viable, LNCaP cells were seeded (5000 cells/well) in three 96-well plates pre-coated with poly-lysine. For each plate, treatments were repeated in triplicate wells and the cells were grown overnight. The cells were then treated with dsRB-SCP/polyIC, polyIC alone or dsRB-SCP alone. The first plate was assayed for survival after 100 hours. The medium in the second plate was changed after 100 hours and survival was assayed after 172 hours. The medium in the third plate was changed after 100 hours and again after 172 hours and survival was assayed after 344 hours.

### Immunoblots

LNCaP cells were seeded in 6-well plates (10^6^ cells/well), grown overnight and treated with dsRB-SCP/polyIC or polyIC alone at the indicated concentrations. After 7, 16 or 24 hours cells were lysed with boiling Laemmli sample buffer (10% glycerol, 50 mmol/L Tris-HCl, pH 6.8, 3% SDS, and 5% 2-mercaptoethanol) and the lysates were then subjected to western blot analysis [75]. The cleavage of PARP and caspase-3 was monitored using anti-PARP (cat#95425), anti-caspase3 (cat#96625) and anti-cleaved caspase-3 (cat#96615) (all from Cell Signaling Technology, Beverly, MA, USA). As an internal control to normalize the amount of protein applied in each lane the blots were also incubated with anti-GAPDH (sc-25778, Santa Cruz Biotechnology, Santa Cruz CA, USA).

### Detection of secreted chemokines (IP-10 and RANTES) by ELISA

LNCaP cells were seeded in 96-well plates in triplicate and grown overnight (10,000 cells/well). Cells were then treated with dsRB-SCP/polyIC or polyIC alone at the indicated concentrations. After 48 hours the medium was collected and the concentrations of IP-10, RANTES and IFN-β were measured using commercial ELISA kits. IP-10 and RANTES were measured using PeproTech ELISA kits from PeproTech (Rocky Hill, NJ, USA) and IFN-β was measured using LumiKine hIFN-β kit (InvivoGen, Toulouse, France).

### RNA isolation, cDNA synthesis and quantitative real-time PCR

LNCaP cells were seeded in 24-well plates (500,000 cells per well) and grown overnight. Cells were then treated for 4 hours with dsRB-SCP/polyIC, polyIC alone or dsRB-SCP alone at the indicated concentrations. The cells were lysed and total RNA was extracted using the EZ-10 DNA Away RNA-Miniprep Kit (Bio Basic, Toronto, Canada). Complementary DNA (cDNA) was synthesized using the High Capacity cDNA Reverse Transcription Kit (Applied Biosystems, Foster City, CA, USA). IFN-β gene expression levels were compared using quantitative real-time PCR and normalized to GAPDH expression using the ΔΔ CT method. The primers for IFN-β quantification were: forward: 5′ ATGACCAACAAGTGTCTCCTCC 3′ and reverse: 5′ GCTCATGGAAAGAGCTGTAGTG 3′. The primers for GAPDH quantification were forward: 5′ GAGCCACATCGCTCAGAC 3′ and reverse: 5′ CTTCTCATGGTTCACACCC 3′.

Chemotaxis of PBMCs LNCaP cells were seeded in 24-well plates pre-coated with poly-lysine (250,000 cells/well) and grown overnight. Then, the medium was replaced by low-serum medium (0.15% FBS) and the cells were treated with dsRB-SCP/polyIC at the indicated concentrations. After 48 hours conditioned medium was collected from the cells and placed in the bottom well of a 24-well Transwell system (microporous polycarbonate membrane (0.5 μm), CorningCostar Corp., Cambridge, MA, USA). Freshly isolated PBMCs (1×10^6^) in low-serum medium (0.15% FBS) were added to the upper chamber. After 3.5 hours, medium from the lower chamber was collected and the migrated cells were quantified by FACS analysis, scatter-gating on lymphocytes.

### Analysis of bystander effects in co-culture systems

In order to measure the viability of a single cell line in co-culture with other cells, we generated cells that expressed luciferase (either LNCaP-Luc/GFP or PC3-Luc/GFP).

The immune-cell-mediated bystander effect was analyzed using LNCaP-Luc/GFP cells co-cultured with PBMCs: LNCaP-Luc/GFP cells were seeded in triplicate in 96-well plates pre-coated with poly-lysine (10,000 cells/well) and grown overnight. The cells were then treated with dsRB-SCP/polyIC, polyIC alone or dsRB-SCP alone at the indicated concentrations. After 24 hours, freshly isolated PBMCs were added to the culture (1×10^5^ per well). 48 hours later, the survival of LNCaP-Luc/GFP cells was measured based on luciferase activity using the Luciferase Assay System.

The combined direct and immune-cell-mediated bystander effect was analyzed using LNCaP cells co-cultured with PC3-Luc/GFP and PBMCs: LNCaP cells were seeded in triplicate in 96-well plates pre-coated with poly-lysine (6,000 cells/well) and grown overnight, and the cells were treated with dsRB-SCP/polyIC, polyIC alone or dsRB-SCP alone. After 16 hours PC3-Luc/GFP cells (4,000 cells/ well) were added to the culture. Eight hours later, i.e. 24 hours after treatment initiation, freshly isolated PBMCs (1×10^5^/ well) were added to the culture. 48 hours later survival of the PC3-Luc/GFP cells was measured based on luciferase activity, using the Luciferase Assay system.

### Tumor spheroid model

Tumor spheroids were generated using agar-coated plates. 96-well plates were coated with 50 μl/well agar (1.5% (wt/vol) dissolved in RPMI) as described by Friedrich, et al. [76]. LNCaP or LNCaP-Luc/GFP cells were seeded (2000 cells per well) and incubated at 37 °C in 5% CO_2_. After 97 hours, a single spherical spheroid 300-400μm diameter had formed in each well.

To measure LNCaP spheroids following treatment with dsRB-SCP/polyIC, we transferred the spheroids individually to 96-well plates (1 spheroid/well) pre-coated with a very thin, even layer of polyHEMA (Sigma, St Louis, MI, USA) (120mg/ml dissolved in 95% ethanol). To transfer the spheroids, we first lifted each spheroid together with its 200 μl of medium into a 96U-well plate (with U-shaped wells). The plate was centrifuged for 10 minutes at 220g and the medium was replaced with 80 μl of fresh medium. The spheroid was then transferred, together with its 80 μl of medium, to the polyHEMA-coated plate. dsRB-SCP/polyIC, polyIC alone or dsRB-SCP alone were added at the indicated concentrations. Treatment continued for 5 days. On days 1, 2, 4 and 5, half of the medium in each well was removed and replaced with fresh medium containing the appropriate treatment. On Day 15, spheroids were stained with calcein AM (1:1000, c3099, Molecular Probes, Eugene, OR, USA) and 0.5μg/ml propidium iodide. Spheroids were monitored using confocal microscopy and size was measured using ImageJ software.

To analyze the immune-cell-mediated bystander effects on tumor spheroids, we treated LNCaP-Luc/GFP spheroids once, directly on the agar plate, with dsRB-SCP/polyIC, polyIC alone or dsRB-SCP alone at the indicated concentrations. After 24 hours fresh PBMCs were labeled using 1 μM CellTracker™ Violet BMQC (Invitrogen, San Diego, CA, USA) according to the manufacturer's protocol. 8×10^4^ PBMCs were added to the spheroid culture. The co-culture was monitored using confocal microscopy

### Statistical analysis

Statistical analyses were performed using unpaired t-test to compare the difference between two group's means. P value indicates the probability of a significant difference between two means, where *P* ≤ 0.05 was considered as a significant difference.

## SUPPLEMENTARY MATERIALS FIGURES


