# Supplementary material for: PSMA-homing dsRNA chimeric protein vector kills prostate cancer cells and activates anti-tumor bystander responses
Source: Oncotarget. 2017 Feb 25;8(15):24046–62. doi: 10.18632/oncotarget.15733 (PMC5421826; doi:10.18632/oncotarget.15733)
Supplement: Supplementary file 1 [file oncotarget-08-24046-s001.pdf]

PSMA-homing dsRNA chimeric protein vector kills prostate cancer cells and activates anti-tumor bystander responses

Supplementary Material

DNA and protein sequences of A. GFP-SCP and B. dsRB-SCP

A.

GFP-SCP DNA sequence: (b.p=1554)
ATGGGCAGCAGCCATCATCATCATCATCACAGCAGCGGCCTGGTGCCGCGCGGCAGCCATAT
GGTGAGCAAGGGCGAGGAGCTGTTCACCGGGGTGGTGCCCATCCTGGTCGAGCTGGACGGCG
ACGTAAACGGCCACAAGTTCAGCGTGTCCGGCGAGGGCGAGGGCGATGCCACCTACGGCAAG
CTGACCCTGAAGTTCATCTGCACCACCGGCAAGCTGCCCGTGCCCTGGCCCACCCTCGTGACC
ACCCTGACCTACGGCGTGCAGTGCTTCAGCCGCTACCCCGACCACATGAAGCAGCACGACTTC
TTCAAGTCCGCCATGCCCGAAGGCTACGTCCAGGAGCGCACCATCTTCTTCAAGGACGACGGC
AACTACAAGACCCGCGCCGAGGTGAAGTTCGAGGGCGACACCCTGGTGAACCGCATCGAGCT
GAAGGGCATCGACTTCAAGGAGGACGGCAACATCCTGGGGCACAAGCTGGAGTACAACTACA
ACAGCCACAACGTCTATATCATGGCCGACAAGCAGAAGAACGGCATCAAGGTGAACTTCAAG
ATCCGCCACAACATCGAGGACGGCAGCGTGCAGCTCGCCGACCACTACCAGCAGAACACCCC
CATCGGCGACGGCCCCGTGCTGCTGCCCGACAACCACTACCTGAGCACCCAGTCCGCCCTGAG
CAAAGACCCCAACGAGAAGCGCGATCACATGGTCCTGCTGGAGTTCGTGACCGCCGCCGGGA
TCACTCTCGGCATGGACGAGCTGTACAAGAAAAGCGGCGGTGGCGGATCCCGTCGTCGCCGT
CGTCGCCGTCGCGGCCGCAAAGCTTCCGCAGAGGTGCAGCTGCAGCAGTCAGGACCTGAACT
GGTGAAGCCTGGGACTTCAGTGAGGATATCCTGCAAGACTTCTGGATACACATTCACTGAATA
TACCATACACTGGGTGAAGCAGAGCCATGGAAAGAGCCTTGAGTGGATTGGAAACATCAATC
CTAACAATGGTGGTACCACCTACAATCAGAAGTTCGAGGACAAGGCCACATTGACTGTAGAC
AAGTCCTCCAGTACAGCCTACATGGAGCTCCGCAGCCTAACATCTGAGGATTCTGCAGTCTAT
TATTGTGCAGCTGGTTGGAACTTTGACTACTGGGGCCAAGGGACCACGGTCACCGTCTCCTCA
GGTGGAGGTGGATCAGGTGGAGGTGGATCTGGTGGAGGTGGATCTGACATTGTGATGACCCA
GTCTCACAAATTCATGTCCACATCAGTAGGAGACAGGGTCAGCATCATCTGTAAGGCCAGTCA
AGATGTGGGTACTGCTGTAGACTGGTATCAACAGAAACCAGGACAATCTCCTAAACTACTGAT
TTATTGGGCATCCACTCGGCACACTGGAGTCCCTGATCGCTTCACAGGCAGTGGATCTGGGAC
AGACTTCACTCTCACCATTACTAATGTTCAGTCTGAAGACTTGGCAGATTATTTCTGTCAGCAA
TATAACAGCTATCCCCTCACGTTCGGTGCTGGGACCATGCTGGACCTGAAATAA

GFP-SCP protein sequence: (AA=516, MW=56823.4)
MGSSHHHHHHSSGLVPRGSHMVSKGEELFTGVVPILVELDGDVNGHKFSVSGEGEGDATYGKLT
LKFICTTGKLPVPWPTLVTTLTYGVQCFSRYPDHMKQHDFFKSAMPEGYVQERTIFFKDDGNYKT
RAEVKFEGDTLVNRIELKGIDFKEDGNILGHKLEYNYNSHNVYIMADKQKNGIKVNFKIRHNIEDG
SVQLADHYQQNTPIGDGPVLLPDNHYLSTQSALSKDPNEKRDHMVLLEFVTAAGITLGMDELYKK
SGGGGSRRRRRRRRGRKASAEVQLQQSGPELVKPGTSVRISCKTSGYTFTEYTIHWVKQSHGKSLE
WIGNINPNNGGTTYNQKFEDKATLTVDKSSSTAYMELRSLTSEDSAVYYCAAGWNFDYWGQGTT
VTVSSGGGGSGGGGSGGGGSDIVMTQSHKFMSTSVGDRVSIICKASQDVGTAVDWYQQKPGQSP
KLLIYWASTRHTGVPDRFTGSGSGTDFTLTITNVQSEDLADYFCQQYNSYPLTFGAGTMLDLK

B.

dsRB-SCP DNA sequence: (b.p=1338)
ATGGGCAGCAGCCATCATCATCATCATCACAGCAGCGGCCTGGTGCCGCGCGGCAGCCATAT
GATGGCTGGTGATCTTTCAGCAGGTTTCTTCATGGAGGAACTTAATACATACCGTCAGAAGCA
GGGAGTAGTACTTAAATATCAAGAACTGCCTAATTCAGGACCTCCACATGATAGGAGGTTTAC
ATTTCAAGTTATAATAGATGGAAGAGAATTTCCAGAAGGTGAAGGTAGATCAAAGAAGGAAG
CAAAAAATGCCGCAGCCAAATTAGCTGTTGAGATACTTAATAAGGAAAAGAAGGCAGTTAGT
CCTTTATTATTGACAACAACGAATTCTTCAGAAGGATTATCCATGGGGAATTACATAGGCCTT
ATCAATAGAATTGCCCAGAAGAAAAGACTAACTGTAAATTATGAACAGTGTGCATCGGGGGT
GCATGGGCCAGAAGGATTTCATTATAAATGCAAAATGGGACAGAAAGAATATAGTATTGGTA
CAGGTTCTACTAAACAGGAAGCAAAACAATTGGCGGCCAAACTGGCCTATCTGCAGATCTTAT
CGGAGAGCGGCGGTGGCGGATCCCGTCGTCGCCGTCGTCGCCGTCGCGGCCGCAAAGCTTCC

GCAGAGGTGCAGCTGCAGCAGTCAGGACCTGAACTGGTGAAGCCTGGGACTTCAGTGAGGAT
ATCCTGCAAGACTTCTGGATACACATTCACTGAATATACCATACACTGGGTGAAGCAGAGCCA
TGGAAAGAGCCTTGAGTGGATTGGAAACATCAATCCTAACAATGGTGGTACCACCTACAATC
AGAAGTTCGAGGACAAGGCCACATTGACTGTAGACAAGTCCTCCAGTACAGCCTACATGGAG
CTCCGCAGCCTAACATCTGAGGATTCTGCAGTCTATTATTGTGCAGCTGGTTGGAACTTTGACT
ACTGGGGCCAAGGGACCACGGTCACCGTCTCCTCAGGTGGAGGTGGATCAGGTGGAGGTGGA
TCTGGTGGAGGTGGATCTGACATTGTGATGACCCAGTCTCACAAATTCATGTCCACATCAGTA
GGAGACAGGGTCAGCATCATCTGTAAGGCCAGTCAAGATGTGGGTACTGCTGTAGACTGGTA
TCAACAGAAACCAGGACAATCTCCTAAACTACTGATTTATTGGGCATCCACTCGGCACACTGG
AGTCCCTGATCGCTTCACAGGCAGTGGATCTGGGACAGACTTCACTCTCACCATTACTAATGT
TCAGTCTGAAGACTTGGCAGATTATTTCTGTCAGCAATATAACAGCTATCCCCTCACGTTCGGT
GCTGGGACCATGCTGGACCTGAAA

dsRB-SCP protein sequence: (AA=446, MW=48614.3)

MGSSHHHHHHSSGLVPRGSHMMAGDLSAGFFMEELNTYRQKQGVVLKYQELPNSGPPHDRRFTF
QVIIDGREFPEGEGRSKKEAKNAAAKLAVEILNKEKKAVSPLLLTTTNSSEGLSMGNYIGLINRIAQ
KKRLTVNYEQCASGVHGPEGFHYKCKMGQKEYSIGTGSTKQEAKQLAAKLAYLQILSESGGGGS
RRRRRRRRGRKASAEVQLQQSGPELVKPGTSVRISCKTSGYTFTEYTIHWVKQSHGKSLEWIGNIN
PNNGGTTYNQKFEDKATLTVDKSSSTAYMELRSLTSEDSAVYYCAAGWNFDYWGQGTTVTVSS
GGGGSGGGGSGGGGSDIVMTQSHKFMSTSVGDRVSIICKASQDVGTAVDWYQQKPGQSPKLLIY
WASTRHTGVPDRFTGSGSGTDFTLTITNVQSEDLADYFCQQYNSYPLTFGAGTMLDLK

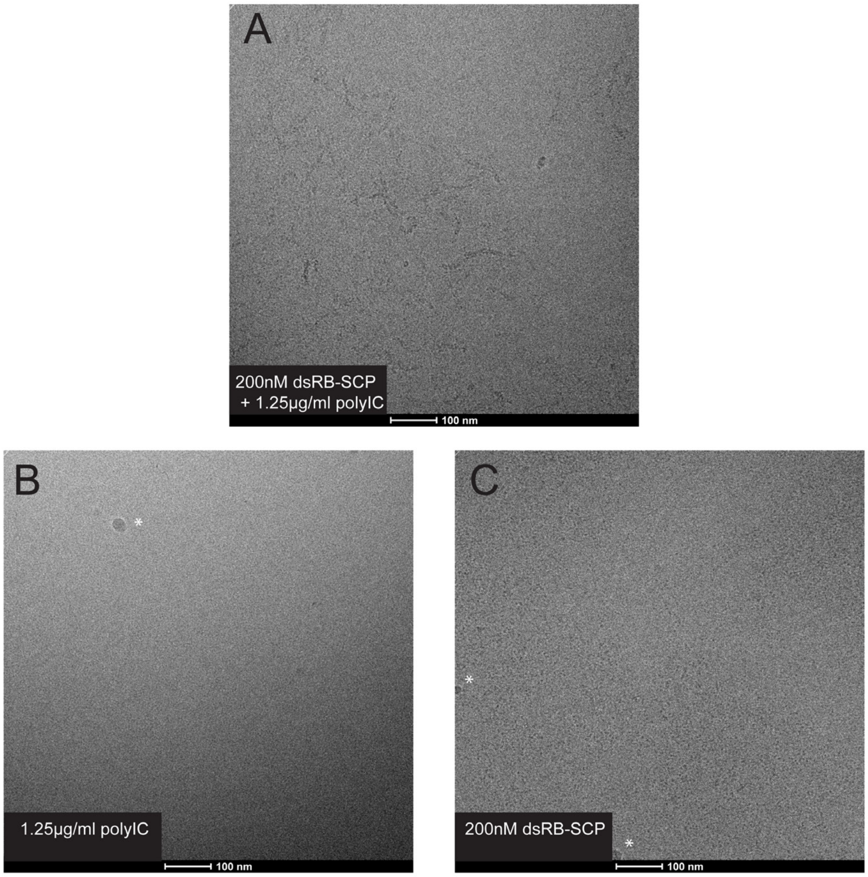

Supplementary Figure 1: Direct imaging of dsRB-SCP/polyIC complex by cryogenic transmission electron microscopy (cryo-TEM). A. The dsRB-SCP/polyIC complexes are visualized as chains of beads on elongated filaments, which are about 114.8 + 34.4 nm in length (mean length) and 8.2 + 3.2 nm wide (mean width) (n=20, measurements were done using ImageJ software). B. PolyIC alone is barely visible as extremely thin threads. C. dsRB-SCP alone is dispersed as tiny dots all over the grid. Asterisks indicate ice on the grid.

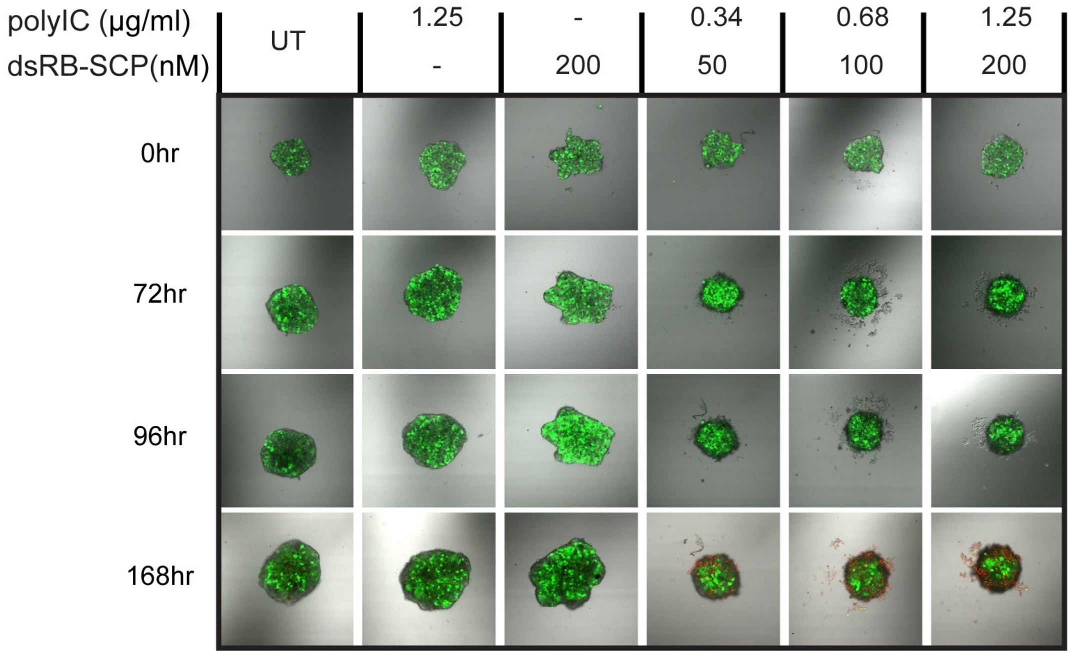

Supplementary Figure 2: Complete time course of treatment with dsRB-SCP/polyIC of LNCaP-Luc/GFP spheroids in the absence of PBMCs. The endpoints only (168 h) are also shown in Figure 6, lower panel. Refer to Figure 6 for detailed description.
